# Understanding Urban Demand for Wild Meat in Vietnam: Implications for Conservation Actions

**DOI:** 10.1371/journal.pone.0134787

**Published:** 2016-01-11

**Authors:** Rachel Shairp, Diogo Veríssimo, Iain Fraser, Daniel Challender, Douglas MacMillan

**Affiliations:** 1 Durrell Institute of Conservation and Ecology (DICE), School of Anthropology and Conservation, Marlowe Building, University of Kent, Canterbury, Kent, CT2 7NR, United Kingdom; 2 School of Economics, University of Kent, Canterbury, CT2 7NP, United Kingdom; University of Regina, CANADA

## Abstract

Vietnam is a significant consumer of wildlife, particularly wild meat, in urban restaurant settings. To meet this demand, poaching of wildlife is widespread, threatening regional and international biodiversity. Previous interventions to tackle illegal and potentially unsustainable consumption of wild meat in Vietnam have generally focused on limiting supply. While critical, they have been impeded by a lack of resources, the presence of increasingly organised criminal networks and corruption. Attention is, therefore, turning to the consumer, but a paucity of research investigating consumer demand for wild meat will impede the creation of effective consumer-centred interventions. Here we used a mixed-methods research approach comprising a hypothetical choice modelling survey and qualitative interviews to explore the drivers of wild meat consumption and consumer preferences among residents of Ho Chi Minh City, Vietnam. Our findings indicate that demand for wild meat is heterogeneous and highly context specific. Wild-sourced, rare, and expensive wild meat-types are eaten by those situated towards the top of the societal hierarchy to convey wealth and status and are commonly consumed in lucrative business contexts. Cheaper, legal and farmed substitutes for wild-sourced meats are also consumed, but typically in more casual consumption or social drinking settings. We explore the implications of our results for current conservation interventions in Vietnam that attempt to tackle illegal and potentially unsustainable trade in and consumption of wild meat and detail how our research informs future consumer-centric conservation actions.

## Introduction

Trade in wildlife poses one of the greatest threats to biodiversity in the tropics [1] and is receiving attention from researchers, conservationists and policymakers alike [2, 3]. Southeast Asia, in particular, is a “hub” for wildlife trade, both legal and illegal [4–6]. Vietnam, for instance, has an illegal trade that generates estimated annual revenues of US$67 million [7].

Vietnam contributes to this trade in several ways. Like other countries in the region, it is a producer and thoroughfare of wildlife traded internationally. It is also, however, a significant consumer of wildlife, particularly wild meat, in urban restaurant settings [8, 9]. To meet this demand, poaching of wildlife is widespread (e.g. [10]) threatening regional and international biodiversity [1, 9, 11].

Generally, interventions to tackle illegal and potentially unsustainable trade in wildlife have focused on limiting supply, typically through the enforcement of regulatory measures [2, 12]. In Vietnam, an array of legislation regulating much of the wild meat supply chain has been enacted. *Decree* 32/2006/ND-CP and *Decree* 82/2006/ND-CP, for example, prohibit the harvest, trade, use and consumption of all species protected under Vietnamese Law. However, ineffective enforcement, the presence of increasingly organised criminal networks and corruption undermine these efforts [7, 13, 14].

As such, the Government of Vietnam promotes captive breeding of wildlife as an alternative solution to the exploitation of wild populations and has established a policy framework regulating the increasing number of commercial wildlife farms that produce a variety of taxa, many of which are consumed as meat [15]. According to economic theory, flooding the market with farmed substitutes for harvested wild species will lower prices and therefore incentives to poach from wild populations [16]. However, doubts have been raised over the appropriateness and efficacy of farming as a conservation tool following findings that wild meat consumers prefer wild-sourced meat over farmed substitutes [17] and findings that wildlife farms continue to launder wild-caught animals [15, 18].

In light of both the limitations of these approaches and the rapidly rising demand for wildlife products [6], attention is turning to demand management and tools such as awareness raising (e.g. [19]) and social marketing [5, 9, 20, 21, 22], which attempt to engineer desirable conservation outcomes e.g., reduced demand. However, for such tools to be effective in changing consumer behaviour an in depth understanding of consumer choices among target audiences is first needed.

In this paper we explore consumer preferences for wild meat and the drivers of consumption among residents of Ho Chi Minh City, Vietnam. We employ a mixed-methods research approach encompassing a hypothetical choice modelling survey, to provide an indication of the relative impact of different attributes of wild meat on consumer choice, and a series of qualitative interviews with wild meat restaurant staff and consumers, to explore the drivers of consumption and consumer choice in depth.

## Material and Methods

### Study site

This research was conducted in Ho Chi Minh City (HCMC), one of several urban centres in Vietnam where wild meat consumption has been quantified. A recent survey found that 50% of sampled HCMC residents had used wild animal products during their lifetime, 75% of which were wild meat [23].

### Ethics statement

Research ethics clearance was obtained from the Research Ethics Advisory Group at the University of Kent. Informed verbal consent was obtained from all respondents prior to participating in the study. Verbal consent was deemed appropriate given the clandestine and sensitive nature of the survey content, and was specifically approved by the institutional ethics board. The date and time of the interview or questionnaire, along with the name of the researcher obtaining consent, were documented prior to commencing the interview or survey. All data collected were anonymous. A business visa was acquired for Vietnam and the research was carried out in association with the Institute of Tropical Biology, part of the Vietnam Academy of Science and Technology, based in Ho Chi Minh City.

### Choice modelling

Choice modelling (also termed a choice experiment) is a survey based stated preference valuation technique that makes use of hypothetical markets to infer respondents’ preferences and demand for goods [24]. A respondent is required to make choices between different hypothetical product alternatives, each described by varying levels of the same attributes, and each providing different levels of utility to the respondent. Consistent with random utility theory and Lancasterian consumer theory, choice modelling is grounded in the assumption that an individual will choose the product alternative whose attributes provide him the greatest utility [25]. It can consequently be used to infer information on which attributes influence consumer choice, the implied ranking of these attributes, and marginal willingness to pay for any increase or decrease in a significant attribute [24]. In our study we used this method to attempt to understand consumer preferences for various attributes of wild meat by presenting respondents with hypothetical menus containing several wild meat products, each defined by different levels of the same product attributes.

The choice survey was a D-efficient Bayesian design produced using Bayesian prior distributions generated from the results of a small-scale pilot [26], also carried out in HCMC. The final design consisted of 10 choice scenarios, which we blocked into two groups of five (A and B). Respondents were randomly assigned to either A or B. Each choice scenario, or ‘menu’, contained three ‘wild meat’ options and a ‘status quo’ option and for each the respondent was asked to choose their preferred option (see S1 Fig for an example of the layout of the choice card).

We selected attributes and their levels (see Table 1) following a review of relevant literature and considering data collected through informal interviews with wild meat consumers and restaurant staff.

**Table 1:** Description of all attributes and their levels chosen for the choice modelling survey (coding is in brackets).

Attribute	Description
Price	Price for enough meat for four persons (VND) divided into five levels: 300,000 (0),
	600,000 (1), 1,100,000 (2), 2,200,000 (3), 4,500,000 (4).
Wild meat-type	Type of wild meat divided into five levels: wild pig (0), deer (1), civet (2),
	king cobra (3), pangolin (4).
Source	Whether the meat was sourced from the wild (0) or from a farm (1).

Price: Price is universally recognised as a primary determinant of demand, with demand falling as prices rises. Nonetheless, there is evidence suggesting that some consumers in Vietnam value and demand wild meat because of its expense [17]. To explore this, we included price as a five level attribute with the levels reflecting the variety in market-prices for different types of wild meat.

The context for the choice task was given careful consideration. As wild meat consumption in Vietnam is a social activity [9], we presented price to respondents as the price for enough meat for them and three ‘friends’. This also allowed us to control for different choices that might be made in different consumption contexts.

Source of the meat: A number of taxa consumed as wild meat are commercially farmed in Vietnam, including deer (*Cervus unicolor–*listed as ‘vulnerable’ on the IUCN red list), soft-shelled turtle (*Pelodiscus sinensis–*listed as ‘vulnerable’ on the IUCN red list), king cobra (*Ophiophagus Hannah–*listed as ‘vulnerable’ on the IUCN red list) and Southeast Asian porcupine (*Hystrix brachyuran–*listed as ‘least concern’ on the IUCN red list) [18]. However, the use of farmed wildlife as a substitute (and as a conservation tool) has been questioned by some conservationists given the apparent preference for wild-sourced meat by consumers [17]. To investigate this further, we included meat source as a binary attribute: wild and farmed.

Wild meat-type: Wild pig (*Sus Scrofa–*listed as ‘least concern’ on the IUCN red list), deer and civet were chosen as they were among the most commonly eaten wild meat-types, as reported in recent surveys [9, 23]. Pangolin (*Manis spp*.*–*all Asian pangolin species are listed as either ‘endangered’ or ‘critically endangered’ on the IUCN red list) and king cobra snake were selected as the fourth and fifth levels because both are consumed for their meat in Vietnam despite being protected under Vietnamese Law and listed in Vietnam’s Red Data Book of threatened species. Respondents also perceived them to be ‘rare’, a characteristic that Drury [17] suggested was important to consumers. This is significant because where a species’ value is tied to its rarity it can result in an extinction vortex as its value rises all the while stocks deplete [27]. Understanding how consumer behaviour responds to perceived rarity is, therefore, important.

331 responses were generated (see Table 2 for a summary of respondent characteristics). Respondents were opportunistically sampled while seated in restaurants and cafes; this was thought to be the only realistic sampling strategy given the clandestine nature of the survey content. In order to target wild meat consumers, each sampled individual was asked if they had ever eaten wild meat. Those who responded affirmatively, and non-consumers who indicated that they would consider consuming wild meat in the future were invited to complete the survey. While our sample was unbalanced in terms of gender, it does reflect the typical urban wild meat consumer as identified in previous studies [9].

**Table 2:** Summary of surveyed respondent characteristics (n = 331).

Variable	Category	Frequency	Percentage
Gender	Male	302	91
	Female	26	8
	Missing	3	1
Age	18–24	24	7
	25–34	171	52
	35–44	101	31
	45–54	22	7
	55–64	9	3
	65 and above	3	1
	Missing	1	0
Attained education	None	1	0
	Primary school	5	2
	Secondary school	69	21
	College	57	17
	Graduate	168	51
	Postgraduate	30	9
	Missing	1	0
Principle occupation	CEO/ director	2	1
	Government official/ police	7	2
	Corporate manager/ team leader/ head of dep.	21	6
	Finance professional	22	7
	Business person	66	20
	Non-finance professional	104	31
	Clerk	1	0
	Service worker	15	5
	Skilled worker	19	6
	Unskilled worker	5	2
	Unemployed	6	2
	Student	15	5
	Retired	6	2
	Missing	42	13

We began our model estimation by employing a Multinomial Logit Model (MNL) to determine aggregate preferences. We then used a Latent Class Model (LCM) to take account of respondent heterogeneity.

#### Model specification

In terms of model specification the random utility model has two parts: an observable deterministic component; and an unobservable random component. We assume a respondent *n* makes one choice from a finite set Z. The utility respondent *n* obtains from selecting an alternative *i* (*i ≠ k*, for all *kϵA*) is:

Uni=βXni+εni (1)

where *U* is the utility obtained by respondent n, *β* is a vector of parameters to be estimated, *X* is a vector of the attributes from the survey and *ε* is the unobservable random component assumed to be a type 1 extreme value distribution. To identify the extent of preference heterogeneity we assume that within the population there are a finite number of segments *S* such that respondent *n* belongs to segment *s (s = 1…*.*S)*. Given this we can re-express the utility that respondent *n* obtains from selecting an alternative *i* as:

Uni|s=βsXni+εni|s (2)

such that the utility parameters are segment specific. The deterministic part of Eq (2) can be divided in two: (i) the specific attributes of the choice made; and (ii) individual specific characteristics (i.e., the socio-economic variables). It follows that the choice probability for respondent *n*, given that they belong to segment *s*, will select an alternative *i* is:

$${\mathrm{Pr}}_{\mathrm{ni}|s}=\left(\frac{e^{\beta_{s}^{\prime }X_{\mathrm{ni}}}}{\sum_{k=1}^{A}e^{\beta_{s}^{'}X_{\mathrm{nk}}}}\right)$$ (3)

Next we use a MNL to place a respondent into a specific segment as follows:

$${\mathrm{Pr}}_{\mathrm{ns}}=\left(\frac{e^{\alpha_{s}^{'}Z_{n}}}{\sum_{s=1}^{S}e^{\alpha_{s}^{'}Z_{n}}}\right)$$ (4)

where *Z*_*n*_ is a vector of respondent-specific variables and *α*_*s*_ a vector of segment specific parameters to be estimated. So, conditional on a specific segment membership, the probability that a respondent selects an alternative *i* is Pr_*ni*_ = Pr_*ns*_Pr_*ni*|*s*_. To estimate the LCM we combine Eqs (3) and (4) as follows:

$${\mathrm{Pr}}_{\mathrm{ni}}=\sum_{s=1}^{S}{\left[\frac{e^{\alpha_{s}^{'}Z_{n}}}{\sum_{s=1}^{S}e^{\alpha_{s}^{'}Z_{n}}}\right]}_{}\left[\frac{e^{\beta_{s}^{\prime }X_{\mathrm{ni}}}}{\sum_{k=1}^{A}e^{\beta_{s}^{'}X_{\mathrm{nk}}}}\right]$$ (5)

Eq (5) is estimated using maximum likelihood estimation requiring that the number of segments *S* be set in advance.

#### Model Selection

We used (i) model log likelihood (LL); (ii) the minimum Akaike Information Criterion (AIC) and (iii) McFaddens Pseudo R^2^ to select the optimal number of segments in the LCM. Results indicated significant model improvement as the number of segments increased. Specifically, the AIC decreased until we had four segments and the rate of change for all criteria (LL, AIC and R^2^) was such that that a four segment LCM fitted the data best (S1 File provides further details and Table A in S1 File provides the results used for segment selection).

### Interviews

Where research can reveal illegal activities, informal, unstructured methods may be more appropriate in terms of accessing data of high internal validity [28, 29]. Accordingly, we conducted interviews (n = 98), largely in an informal or unstructured format [30], in addition to and alongside the hypothetical choice modelling survey.

Interviewees were sampled on completion of the survey, or, during opportunistic visits to restaurants selling wild meat. For a breakdown of sampling strategies, interview types and respective sample sizes see Table 3. Interviews were conducted with individuals or groups and some respondents were interviewed more than once. All interviews were conducted with the aid of a pre-prepared guide, however minimal structuring was provided by the researcher. Questions were open-ended and neutral and intended to elicit original and undirected responses from interviewees. Interviews were completed in Vietnamese or English, according to the interviewee’s preference and lasted between 10–30 minutes. Due to the sensitive nature of the subject-matter interviews were not recorded, rather, notes were made during the interview.

**Table 3:** Summary table of qualitative data collection styles, associated sampling strategies and sample sizes.

Sampling strategy	Interview style	Interest group	Number of interviews	Number of interviewees
Opportunistic	Informal	Wild meat restaurant staff (HCMC)	10	10
		Wild meat consumers	29	30
	Unstructured	Ex-wild meat restaurant staff (HCMC)	2	3
		Wild meat restaurant staff (not HCMC)	2	2
		Wild meat consumers	44	50
		Wildlife trader	1	1
Targeted	Unstructured	Wild meat consumers	2	2
	Semi-structured	Wild meat restaurant staff	7	4
		Wild meat consumers	1	1
		Total:	98	103

Analysis followed an iterative approach; themes were not predefined but were identified as they emerged from the data [29, 31]. Moreover, it took into account the fact that translation seldom conveys exact equivalence in meaning [28]. As such, quotes do not claim to be a precise translation but nonetheless reflect the original meaning as much as possible. Quotes we present portray the main emerging themes and are coded: RS–wild meat restaurant staff; FRS–former wild meat restaurant staff; C–respondent has eaten wild meat.

## Results

### Choice modelling results

We report the MNL (Table 4), which indicates aggregate preferences, and a four segment LCM (Table 5).

**Table 4:** Multinomial Logit Model (MNL) estimates for the hypothetical choice modelling survey. Where statistically significant, the coefficients show increases or decreases in utility on the average respondent for changes in each attribute level away from the baselines described in Table 2. For the dummy coded wild meat types, pangolin is taken as the excluded level.

Attribute	Coefficient	SE
Wild pig	1.133***	0.112
Deer	0.634***	0.101
Civet	-0.002	0.125
King cobra	0.404***	0.105
Source	-0.338***	0.073
Price	-0.454***	-0.03
ASCSQ	-1.029***	0.112

SE = standard error; significance levels are represented by asterisks (***P<0.001, **P<0.01, *P<0.05); ASCSQ = status quo alternative specific constant

**Table 5:** Latent Class Model (LCM) estimates for the hypothetical choice modelling survey. Where statistically significant, the coefficients show increases or decreases in utility provided by changes in attribute levels away from the baselines shown in Table 2. For the dummy coded wild meat types, pangolin is taken as the excluded level.

	Segment 1		Segment 2		Segment 3		Segment 4	
Attributes	Coefficient	SE	Coefficient	SE	Coefficient	SE	Coefficient	SE
Wild pig	5.473***	1.522	5.441***	1.38	3.38***	0.491	-1.009***	0.341
Deer	4.373***	1.415	0.758	0.727	2.683***	0.441	-1.124***	0.32
Civet	-25.963	44.2	1.563**	0.635	0.679	0.436	-0.343	0.29
King cobra	1.393	1.244	2.522***	0.826	1.893***	0.432	-0.534**	0.246
Source	0.324	0.489	-5.282***	1.159	-0.378**	0.166	-0.005	0.22
Price	-2.618***	0.612	-1.338***	0.331	-0.442***	0.058	-0.641***	0.072
Education	1.629***	0.547	0.628	0.583	-0.295	0.331		
Occupation	1.323***	0.457	0.291	0.531	-0.001***	0.001		
ASCSQ	1.974	1.358	-5.757***	1.356	0.276	0.477	-3.003***	0.391

SE = standard error; significance levels are represented by asterisks (***P<0.001, **P<0.01, *P<0.05); ASCSQ = status quo alternative specific constant

The four segments of the LCM each captured different aspects of respondent behaviour. In all segments price was negative and statistically significant indicating a preference for the lower priced menu options. Source was statistically significant in segments two and three with the negative coefficient in both segments indicating a preference for wild as opposed to farm-sourced wild meat. Summing the probability of class membership, segments two and three accounted for approximately 55% of respondents, thus the source of the meat mattered to the majority of respondents. In terms of the meat-types, pig, deer and king cobra were generally preferred (relative to pangolin), except segment four where pangolin was the most-preferred.

In terms of segment membership (reported for three segments due to an adding-up restriction required for model identification) the best model specification included dummy variables for education and occupation. We found that segment 1 members are associated with higher education levels but lower status occupation-types. (This counter-intuitive finding may reflect difficulties associated with collecting data, particularly personal data, on sensitive and potentially illegal subjects. All results must, therefore, be interpreted with this in mind). Membership of segment 3 is associated with higher status occupation-types. Members of this latter group were likely to prefer wild-sourced wild meat.

Finally, we were able to estimate marginal effects for the preferred LCM specification. The marginal effect tells us by how much the probability of a choice changes given a unit change in price. Thus it is akin to ‘price elasticity of demand’ (which could not be estimated due to the type of model specification being employed). Considering the price attribute in detail we found the marginal effect to be -0.15, which indicates that choice was very price inelastic.

### Qualitative results

#### Price

Interview data confirmed that price was a key aspect of decision making. Interviewees considered lower-priced wild meat a suitable or novel selection in more casual, recreational consumption or drinking contexts:

“I see it on the menu, strange meats, at an acceptable price, and I want to try them” (C)

“*For what reasons do you eat wild meat*? To try because my friends tell me it’s good. Me and my friends eat it on the weekend when we go out drinking. When I go out drinking I choose between seafood and wild meat, it’s a habit now” (C)

Contrastingly, more expensive wild meat-types were associated with wealthy and high-ranking individuals who consume it to communicate their own status and wealth:

“Rich people (eat soft-shelled turtle) to show their high class because (soft-shelled turtle) is expensive” (FRS).

Equally, interviewees described how expensive wild meat-types are purchased to convey the status of, and therefore, respect of others. For this reason they are widely used in business to promote good business-relations and aid the contract-signing process. This, reported interviewees, “is the business culture” (C).

“Pangolin has the highest price that’s why you eat it with business contacts it’s a question of respect, well not really respect but about showing the status of the invited persons and the invitees” (C)

Expensive wild meats were also considered an effective “diplomatic” (C) tool, used to secure favours from those in influential positions. Interviewees explained that in these consumption contexts, not only is high cost a symbolically important product characteristic, but, it is also inconsequential as they have access to corporate or institutional funds. One consumer explained how:

“It doesn’t matter about the price I can order as high as I like the company pays” (C)

Others indicated that even if they had to pay out of their own funds, still the cost would be insignificant in light of what they stand to gain:

“They do not care about the money they spend on the meal because what they pay they will gain back” (RS)

#### Wild meat-type

Interviewees reported that the rarer and more expensive wild meats are eaten by those situated towards the top-end of society.

“The price of pangolin is 10 times higher than civet so there must be a difference in the income of the people who eat these different meats. The people who eat pangolin can throw money at you and make you die. The people who eat civet have an income a little higher than medium the people who eat king cobra have an income between those who eat civet and pangolin” (FRS)

*What about bear or pangolin*? “Only my boss and company managers would eat that” (C)

Interviewees also associated different wild meat-types with different consumption contexts, and linked the consumption of rarer and more expensive wild meat-types with lucrative business or institutional contexts:

“*Which wild meats would you eat with friends*? Ones that are not very expensive and rare. For close friends I would choose the same as I would choose with business contacts, strange ones which are quite expensive and rare” (C)

“For friends soft-shelled turtle and cobra snake we usually hang out and the most important thing is fun. For business pangolin and civet they are the highest quality” (C)

Interviewees indicated that underlying this heterogeneity is a value attached by certain consumers to those wild meat-types which are expensive and rare. These product characteristics render wild meat suitable for displays of personal wealth and status, and also, for showing the wealth and status of others.

“I have to choose the most expensive to show my respect to them” (C)

“It is a Vietnamese belief that the more rare the food is the more respect you show to your business contacts” (C)

#### Source of the meat

In agreement with the results of the choice modelling interviewees expressed a widespread preference for wild as opposed to farm-sourced wild meat and considered the farmed substitute inferior. Interviewees distinguished between active wild animals which eat a natural diet and inactive farmed animals which eat an artificial and chemically enhanced diet. This distinction means that wild-sourced meat is perceived as superior in terms of its: (i) quality, (ii) nutritional value, (iii) health benefits (reflecting aspects of traditional Chinese medicine philosophy) and (iv) taste. Additionally, the perceived aphrodisiac abilities of wild-sourced wild meat were alluded to by many interviewees.

Interviewees also mentioned a perceived link between the source of wild meat and its exclusivity, rarity and cost. Wild animals are perceived as more rare and expensive than farmed substitutes. They are therefore symbolically more appropriate for demonstrating wealth and status.

“Everyone knows that wild civet has a high value…so when they want to show their value they eat it. But farmed civet, everyone can have it, so its value is not high… so it does not show their high value” (C)

For these reasons, for certain high-status individuals and in some (lucrative) business consumption contexts the farmed substitute was considered unacceptable.

“If you take them [business contacts] to a wild meat restaurant, if it is not wild wild meat, it is like you don’t respect them, like somehow you have tricked them” (C)

On the other hand, and in spite of a general preference for the wild product, some interviewees explained that they do eat farmed equivalents: they are more affordable, suitable for casual consumption or drinking contexts, and buying the farmed product means that the consumer avoids being cheated by restaurants which, according to informants, do sell farmed substitutes under the guise of being wild.

“The best solution is to order farmed because the price is so different so then you will be sure that you are not being tricked” (C)

## Discussion

Over the last few decades the Vietnamese government has committed to tackling the illegal and potentially unsustainable consumption of species for their meat by enacting legislation that regulates the harvest, trade, and consumption of wildlife, and by promoting the captive breeding of consumed species. Recent years have also seen an increased focus on various ‘demand reduction’ strategies [19–22]. While all have likely had some impact, the illegal consumption of wild meat (and other wildlife) remains a problem. Here we explore the implications of our results for conservation actions noting from the outset that this was an exploratory case study, with relatively small sample sizes, and which employed a targeted sampling strategy. Results are not, therefore, generalizable to the population as a whole.

### Regulation

Enacting and enforcing regulation is a vital component of strategies to address wildlife crime and recent seizure data (e.g., [32]) attest to its impact. Nonetheless, our results suggest that the role of regulation in this context is likely to be impeded by the following factors.

First, and consistent with the findings of related research [9, 17], our results indicate that high-status individuals and business people purposefully select rarer, wild sourced and more expensive wild meats to secure business and social advantage. In order to fully understand the extent of the social benefits conferred by wild meat, it is enlightening to consider the broader socio-cultural context of consumption. In collectivist Asian cultures, a society is typically very hierarchical and its individuals preoccupied with their own position within it. Accordingly, displays of social status and ‘face’ (an individual’s self-value [33]) are common, and are reinforced by a cultural inclination to conform to expected behaviours [34, 35]. Like other luxury goods, wild meat appears to be appropriate for conveying status and face in a hierarchically conscious society, in this case Vietnam. It may also be the case that the perceived differences in the value of distinctive wild meat-types provide the consumer with the opportunity to assert inter-group differences in status and face [35].

The social and financial benefits conferred by consuming wild meat and the apparent relationship between wild meat and the politico-business nexus will make enforcement difficult. This will be particularly so where these individuals or institutions are willing and able to pay rising prices and where they have the funds or political leverage to avoid prosecution.

Second, wild meat is considered more attractive for communicating status and hence more valuable in a business setting if the species consumed are rare and expensive. This is significant because where a species is valued for its rarity and high price it can become trapped in an extinction vortex as its value will continue to rise as its stock in the wild deplete [27]. It is also important because it suggests that a reduction in supply due to enforcement activities may, *ceteris paribus*, increase the perceived rarity of certain wild meat-types among some consumers, potentially rendering them more appealing [14].

### Farming

According to economic theory, farmed substitutes for harvested wild species will lower prices and thereby incentives to poach wild populations [16]. However, our results suggest that farmed animals may only act as effective substitutes in certain circumstances.

While the results of our choice modelling reflect a widespread preference among the sample for wild-sourced wild meat, our qualitative data suggest that farmed substitutes are satisfactory in more casual eating or drinking contexts, and among consumers attracted by their lower prices. However, two important points must be noted here. First, even where farmed animals are satisfactory substitutes, there remains a risk to wild populations as costs associated with farming create incentives to launder wild animals. This has been widely reported [36, 15, 18, 37]. Second, it is entirely possible that farmed wild meat is creating a new market, attracting new consumers, rather than displacing existing demand for wild animals. This issue was not explored here, but warrants further investigation.

For a ‘super-elite’ segment of Vietnamese society, whose members consume wild sourced animals to convey status and wealth, farmed sourced wild meat is not an appropriate substitute as it lacks the product characteristics needed to symbolically convey status and wealth–expense and rarity. Indeed, it is possible that the availability of substitutes in the market is causing these consumers to place increased emphasis on finding the rarer, wild specimens to assert inter-group differences in status and face. Where combined with a willingness and ability to pay rising prices, this could incentivize the exploitation of the last rare, wild individuals of farmed species [27], or alternatively, shift demand to those species whose biology precludes their being farmed.

Of future significance here is Vietnam’s booming economy–between 1990 and 2010 it grew at an average annual rate of 7.3%, and the per capita income almost quintupled [38]. It may therefore be anticipated that demand for highly-valued, rare, wild-sourced wild meat may also increase as the emerging ‘super-elite’ seek consumption as a means of demonstrating their economic achievement and high societal position.

It is additionally worth noting that where demand is driven by the perceived health and nutritional benefits of eating wild-sourced wildmeat, which reflect aspects of Chinese medicinal philosophy, farmed substitutes are also unlikely to be considered satisfactory substitutes.

### Consumer-centric campaigns

Recent literature advocates the use of consumer-targeted campaigns (e.g. awareness raising or social marketing) to shift behaviour and reduce demand for illegal wildlife products [9, 14, 20]. However, for this to be a realistic conservation option a greater understanding of the consumer and the drivers of consumption is needed in order to be able to create effective messaging and target the right audience.

Results from our exploratory study indicate that effecting behaviour change in this context will not be easy. A successful campaign will have to approach the issue in a culturally-grounded way and overcome the following barriers to behaviour change: the role of wild meat consumption in communicating status, its perceived health benefits, and the apparent relationship between wildmeat and the politico-business nexus. It will also need to be highly targeted and account for heterogeneous demand (e.g. see [22]).

None of these barriers to change are, however, an excuse for inaction and many of them do in fact present opportunities for leveraging behaviour change. For instance, given consumers’ preoccupations with meat-related health concerns, any campaign may do well to emphasise the potentially detrimental health implications of consuming unregulated, wild and illegally sourced meat. Campaigns that seek to undermine wild meat’s role as a status symbol might also be a good starting point.

Looking ahead, we suggest that further research is needed to understand not only consumer decisions about wild meat but also how to integrate behaviour change into the broader socio-cultural and institutional landscape. Equally important will be understanding the media and information sources that are typically used and that are trusted and esteemed by target audience members (e.g. [8]), as well as the type and form of message that is likely to produce changes in behaviour [14].

## Supporting Information

S1 Fig Example of the layout of the choice cards or ‘menu’ presented to respondents as part of the hypothetical choice modelling survey. (DOCX)

S1 File Model selection. Table A in S1 File. Model comparison results.
(DOCX)
