# Supplementary material for: Understanding Urban Demand for Wild Meat in Vietnam: Implications for Conservation Actions
Source: PLoS One. 2016 Jan 11;11(1):e0134787. doi: 10.1371/journal.pone.0134787 (PMC4709058; doi:10.1371/journal.pone.0134787)
Supplement: S1 Fig — (DOCX) [file pone.0134787.s001.docx]

| **A**  <Drawing of pangolin>  Pangolin  2.200.000 VND / 4 persons  Farmed | **B**  <Drawing of king cobra snake>  King cobra  1.100.000 VND / 4 persons  Farmed | **C**  <Drawing of  civet>  Civet  4.500.000 VND / 4 persons  Wild | **D**  None of these options  Alternative, non-wildmeat menu  280.000 VND / 4 persons |
| --- | --- | --- | --- |
