# Supplementary material for: Understanding Urban Demand for Wild Meat in Vietnam: Implications for Conservation Actions
Source: PLoS One. 2016 Jan 11;11(1):e0134787. doi: 10.1371/journal.pone.0134787 (PMC4709058; doi:10.1371/journal.pone.0134787)
Supplement: S1 File — Table A in S1 File. Model comparison results. (DOCX) [file pone.0134787.s002.docx]

**S1. Supplementary information: model selection.**

We began our analysis by examining how our model performed as we increased the number of segments in the LCM. Our results for segment selection are shown below (Table A).

As would be expected the log likelihood and McFadden’s Pseudo R^2^ increased as we went from the MNL to the LCM and then as we increased the number of segments. This occurred until we went from four to five segments. This indicated that there was heterogeneity in the data but that we needed no more than 4 segments.

When we then considered the other model selection criteria reported in Table 5 (i.e. AIC, BIC and AIC3) we can see that the results indicated significant model improvement as the number of segments increased. Specifically, we can see that the AIC decreased until we had four segments. For BIC the improvement from 3 to 4 segments was much bigger than for 4 to 5. For AIC3 the magnitude of improvement was also greatest for 3 to 4 segments compared 4 to 5. The rate of change for all criteria (AIC, BIC and AIC3) was such that we can see that the 4 LCM fitted the data best. To ensure that this choice of model specification was sensible we then examined the model specific results where we found that the LCM4 yielded consistent and interesting results.

**Table A. Model comparison results.**

| Model | K | LL | AIC | BIC | AIC3 | Pseudo R^2^ |
| --- | --- | --- | --- | --- | --- | --- |
| MNL | 7 | -2029.6 | 4073.2 | -2003.7 | 4017.2 | 0.12 |
| LCM2 | 17 | -1902.9 | 3839.8 | -1839.9 | 3703.8 | 0.17 |
| LCM3 | 27 | -1804.9 | 3663.8 | -1704.8 | 3447.8 | 0.21 |
| LCM4 | 37 | -1754.9 | 3583.1 | -1617.8 | 3287.8 | 0.23 |
| LCM5 | 47 | -1778.2 | 3650.5 | -1604.0 | 3274.4 | 0.225 |

K (number of parameters estimated); N (sample size); LL (model log likelihood); AIC (Akaike’s information criterion); BIC (Bayesian information criterion); AIC3 (Modified Akaike’s information criterion with 3 as penality factor); Pseudo R^2^ (the value of the LL for a constant only model).
